# Instability of the Octarepeat Region of the Human Prion Protein Gene

**DOI:** 10.1371/journal.pone.0026635

**Published:** 2011-10-19

**Authors:** Baiya Li, Liuting Qing, Jianqun Yan, Qingzhong Kong

**Affiliations:** 1 Department of Physiology and Pathophysiology, Xi'an Jiaotong University School of Medicine, Xi'an, Shaanxi, China; 2 Department of Pathology, Case Western Reserve University, Cleveland, Ohio, United States of America; Consejo Superior de Investigaciones Cientificas, Spain

## Abstract

Prion diseases are a family of unique fatal transmissible neurodegenerative diseases that affect humans and many animals. Sporadic Creutzfeldt-Jakob disease (sCJD) is the most common prion disease in humans, accounting for 85–90% of all human prion cases, and exhibits a high degree of diversity in phenotypes. The etiology of sCJD remains to be elucidated. The human prion protein gene has an octapeptide repeat region (octarepeats) that normally contains 5 repeats of 24–27 bp (1 nonapeptide and 4 octapeptide coding sequences). An increase of the octarepeat numbers to six or more or a decrease of the octarepeat number to three is linked to genetic prion diseases with heterogeneous phenotypes in humans. Here we report that the human octarepeat region is prone to either contraction or expansion when subjected to PCR amplification *in vitro* using Taq or Pwo polymerase and when replicated in wild type *E. coli* cells. Octarepeat insertion mutants were even less stable, and the mutation rate for the wild type octarepeats was much higher when replicated in DNA mismatch repair-deficient *E.coli* cells. All observed octarepeat mutants resulting from DNA replication in *E.coli* were contained in head-to-head plasmid dimers and DNA mfold analysis (http://mfold.rna.albany.edu/?q=mfold/DNA-Folding-Form) indicates that both DNA strands of the octarepeat region would likely form multiple stable hairpin structures, suggesting that the octarepeat sequence may form stable hairpin structures during DNA replication or repair to cause octarepeat instability. These results provide the first evidence supporting a somatic octarepeat mutation-based model for human sCJD etiology: 1) the instability of the octarepeat region leads to accumulation of somatic octarepeat mutations in brain cells during development and aging, 2) this instability is augmented by compromised DNA mismatch repair in aged cells, and 3) eventually some of the octarepeat mutation-containing brain cells start spontaneous *de novo* prion formation and replication to initiate sCJD.

## Introduction

Prion diseases, or transmissible spongiform encephalopathies (TSEs), are a unique family of fatal neurodegenerative diseases that affect both humans and animals. Prion replication requires the conformational conversion of the cellular prion protein (PrP^C^) from an alpha-helical conformer to beta-sheet rich disease-associated aggregates (PrP^Sc^). Human prion diseases include Creutzfeldt-Jakob disease (CJD), fatal insomnia, Gerstmann-Sträussler-Scheinker disease (GSS), Kuru, and the newly identified variably protease-sensitive prionopathy (VPSPr) [1], [2]. Human prion diseases can be grouped into three classes based on etiology: familial (genetic), sporadic, and acquired (infectious), among which sporadic CJD (sCJD) is the most common, accounting for 85–90% of all human prion cases with very diverse phenotypes. However, practically nothing is known about the mechanisms underlying the development of sCJD.

**Figure 1:**
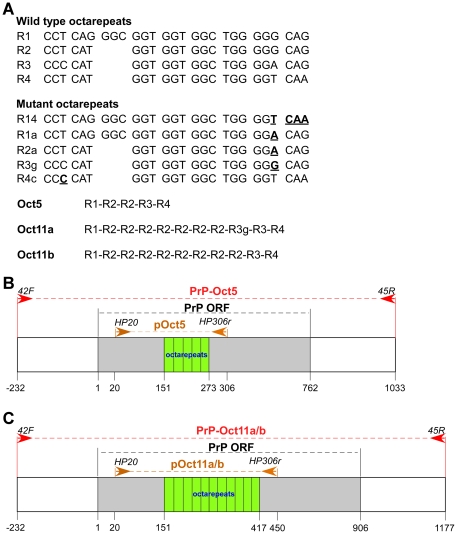
Human *PRNP* octarepeat sequences and cloned octarepeats for instability analysis. (A) Wild type and mutant human octarepeat sequences. In the mutant octarepeats, the mutated bases are in bold case and underlined. R14 could be a chimera repeat between R1 and R4; R1a could be a chimera repeat between R1 and R3; R2a could be a chimera between R2 and R3. The repeats in pOct5, pOct11a and pOct11b are listed. (B) Diagram of cloned wild type human *PRNP* octarepeats used for instability analysis. PrP-Oct5: a region encompassing the wild type PrP ORF (762 bp), 232 bp upstream non-coding sequence and 271 bp downstream non-coding sequence subcloned into pGEM-T after PCR amplification (template: wt human genomic DNA, primers: 42F and 45R). pOct5: the wild type octarepeat region subcloned into pGEM-T after PCR amplification (template: PrP-Oct5, primers: HP20 and HP306r). Arrows denote the primers. (C) Diagram of cloned insertion mutant human *PRNP* octarepeats used for instability analysis. PrP-Oct11a or PrP-Oct11b: a region encompassing an 11-repeat mutant PrP ORF (906 bp), 232 bp upstream non-coding sequence and 271 bp downstream non-coding sequence subcloned into pGEM-T after PCR amplification (template: one of two human genomic DNA samples containing different 11-repeat octarepeats, primers: 42F and 45R). pOct11a or pOct11b: the 11-repeat octarepeat region subcloned into pGEM-T after PCR amplification (template: PrP-Oct11a or PrP-11b, primers: HP20 and HP306r). Arrows denote the primers.

The human prion protein (PrP) is encoded by the *PRNP* gene, a single copy gene on chromosome 20. A large number of point mutations in the *PRNP* coding region have been linked to inherited prion diseases with diverse phenotypes: familial CJD (fCJD), GSS, fatal familial insomnia (FFI), and mixed phenotypes [1]. The most common *PRNP* point mutations include E200K (fCJD), P102L (GSS), D178N-129M (FFI), and D178N-129V (fCJD) [1]. In addition, *PRNP* has an octarepeat region (R1-R2-R2-R3-R4), of which the R1 repeat encodes a nonapeptide (PQGGGGWGQ) and the other four repeats all encode octapeptides (PHGGGWGQ) (Figure 1A). Insertion mutations with 1–9 extra octarepeats or deletion mutations with loss of two octarepeats also cause familial prion diseases, in which the clinical and pathological phenotypes are heterogeneous and heavily influenced by the number of octarepeats [1]. Some of the insertion mutants contain novel variant repeats (Figure 1A) that may have resulted from recombination between wild type repeats [3]. These pathogenic *PRNP* mutations are believed to cause prion diseases by rendering the corresponding mutant PrP protein more prone to adopting a prion-associated conformation [4], [5].

Somatic mutations of specific gene(s) have been reported to be critical for the development of cancer [6], [7] and neurodegenerative diseases such as Huntington's disease [8]–[10] and Alzheimer's disease [11]. In Huntington's disease patients, the CAG repeats of the huntingtin gene expand in an age-dependent manner in brain tissues [12], especially the striatum, leading to the formation of huntingtin protein aggregates in the cytoplasm [13]. Somatic mutation of the presenilin-1 gene was also found in an early-onset sporadic Alzheimer patient who exhibited somatic mosaicism for the mutation in peripheral lymphocytes and cerebral cortex [11].

We hypothesize that the octarepeat region of the human *PRNP* gene is unstable in somatic cells, and that somatic octarepeat mutations accumulate in long-living neurons and/or other cells in the brain during development and/or aging, which may result in spontaneous *de novo* formation of infectious prions in some brain cells containing mutated octarepeats to initiate sCJD. The highly heterogeneous phenotypes associated with familial octarepeat mutations [1] could explain the diversity of sCJD. Here we report that the human *PRNP* octarepeat sequence is unstable during PCR amplification *in vitro* or DNA replication in *E.coli* and that DNA mismatch repair deficiency leads to higher rates of octarepeat mutation in *E.coli*. These findings are consistent with a somatic octarepeat mutation-based etiology for sCJD in humans.

## Results

### Mutation of the octarepeats during PCR amplification

The wild type octarepeat region of human *PRNP* was cloned by PCR amplification from a subject with a wild type *PRNP* gene (Figure 1B). The wild type PrP ORF was amplified with Taq polymerase and primers 42F and 45R to obtain a PCR fragment that contained 232 bp of 5′ non-coding sequence, the wild type PrP ORF (762 bp) and 271 bp of 3′ non-coding sequence, which was subsequently cloned into the pGEM-T vector to obtain PrP-Oct5. Similarly, two 11-repeat mutant PrP ORFs were PCR amplified from two subjects with different 11-repeat octarepeats, which were then cloned into pGEM-T to obtain PrP-Oct11a and PrP-Oct11b (Figure 1C). Sequencing of DNA isolated from single colonies indicated that the PrP-Oct5 clone contained the wild type R1-R2-R2-R3-R4 octarepeats, the PrP-Oct11a clone contained the R1-(R2)^7^-R3g-R3-R4 octarepeats and the PrP-Oct11b clone contained the R1-(R2)^8^-R3-R4 octarepeats (Figure 1A).

**Figure 2:**
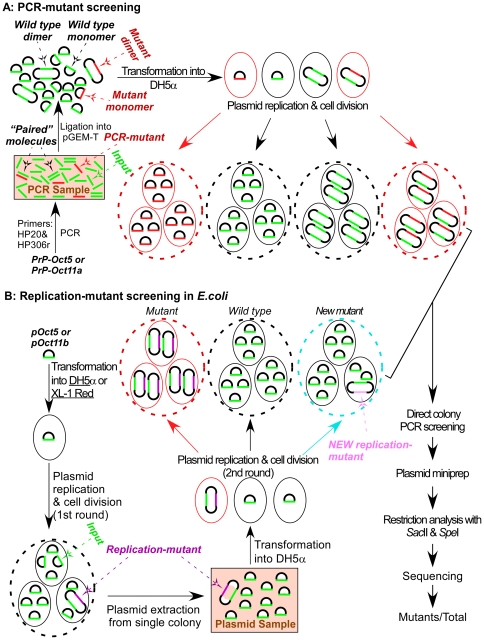
Schematic diagrams for analysis of octarepeat mutation rate during PCR or replication in *E.coli*. (A) Measurement of octarepeat mutants resulting from PCR amplification of octarepeats. Cloned PrP ORF sequences (PrP-Oct5 and PrP-Oct11a) were subjected to PCR with primers HP20 and HP306r. The PCR products (depicted in a shaded box), which contained input (green line) and PCR-mutant (red line) octarepeat sequences as well as “paired” molecules (two parallel lines), were cleaned up and ligated to the pGEM-T vector, producing 4 kinds of ligation products: mutant monomer, wild type monomer, wild type dimer, and mutant dimer. The ligation products were transformed into DH5α competent cells, and the resulting colonies were directly examined by PCR with primers HP50f and HP293r. Plasmid DNAs were extracted from colonies containing mutant octarepeats, subjected to restriction analysis with *Sac*II and *Spe*I, and sequenced. The number of mutant colonies over the total number of colonies screened was calculated to represent the octarepeat mutation rate during the PCR process. Small oval: individual *E.coli* cell; big dashed-line oval: *E.coli* colony. (B) Measurement of octarepeat mutants resulting from DNA replication in *E.coli*. pOct5 or pOct11b was used to transform competent DH5α or XL-1 Red *E.coli* cells, and plasmid DNA sample prepared from a single colony (depicted in a shaded box) was used to transform competent DH5α cells. The mutant colonies (containing only mutant plasmid, red dashed-line oval) and new mutant colonies [containing some cells with the NEW replication-mutant octarepeat insert (light purple), light blue dashed-line oval] were screened out as in (A). The number of mutant colonies (excluding the new mutant colonies) over the total number of colonies examined should reflect the octarepeat mutation rate during the 1^st^ round of plasmid replication and cell division in *E.coli* (DH5α or XL-1 Red).

**Figure 3:**
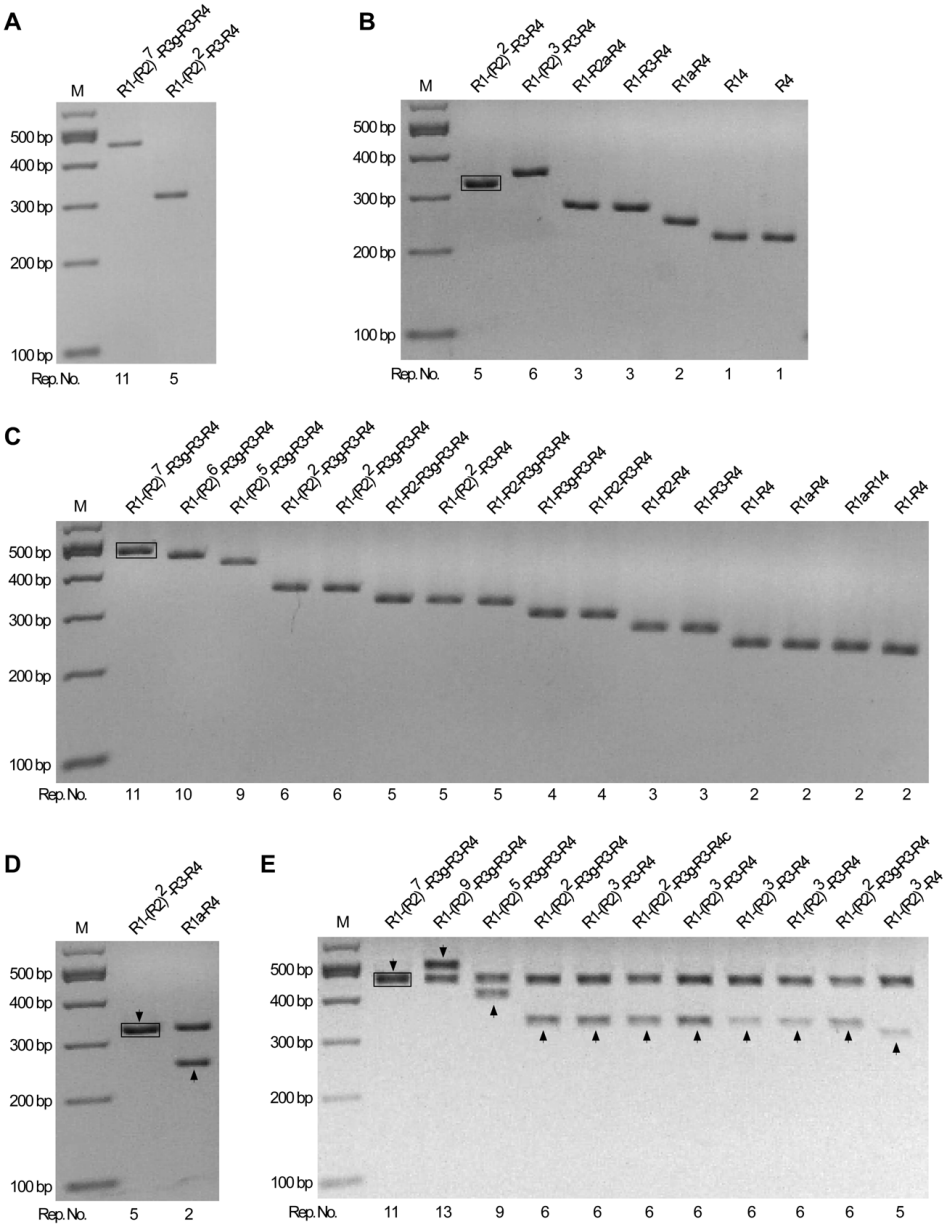
Instability of octarepeats during PCR amplification by Taq Polymerase. (A) PCR products from the PrP-Oct5 and PrP-Oct11a templates. The octarepeat regions PCR amplified by Taq polymerase from PrP-Oct5 and PrP-Oct11a with primers HP20 and HP306r were cleaned up and separated on a 2% agarose gel. (B) Mutant octarepeat clones from PCR amplification of the PrP-Oct5 template: restriction analysis with *Sac*II and *Spe*I. Six mutant clones and one wild type clone are shown. The black box marks the template-sized Oct5 band from a wild type clone. (C) Mutant octarepeat clones from PCR amplification of the PrP-Oct11a template: restriction analysis with *Sac*II and *Spe*I. Same as in (B) except that PrPOct11a was the template. Fifteen mutant clones and one wild type clone are shown. The black box marks the template-sized Oct11 band from a non-mutant clone. (D) A mutant octarepeat clone containing two octarepeat inserts from PCR amplification of PrP-Oct5. *Sac*II and *Spe*I digestion of this mutant clone produced two octarepeat inserts; one was the 5-repeat wild type Oct5 while the other was a 2-repeat deletion mutant (R1a-R4). The arrowhead points to the band whose sequence is shown above the lane. The black box marks the template-sized Oct5 band from a non-mutant clone. (E) Mutant octarepeat clones containing two octarepeat inserts from PCR amplification of PrP-Oct11a. *Sac*II and *Spe*I digestion of the 10 mutant clones produced two octarepeat inserts; one was the 11-repeat parental Oct11a in all clones while the other was a mutant octarepeat sequence of varying sizes and sequences. The arrowhead points to the band whose sequence is shown above the lane. The black box marks the template-sized Oct11 band from a non-mutant clone. For all panels, the octarepeat sequence is indicated above each lane; Rep. No., number of repeats; M,100-bp DNA Ladder.

The instability of the octarepeat region during PCR amplification was evaluated by determining the percentage of mutant molecules in the PCR products (Figure 2A). First, the octarepeat region was amplified from the cloned PrP-Oct5 or PrP-Oct11a as templates using Taq polymerase and primers HP20 and HP306r. Analysis of the PCR products on a 2% agarose gel demonstrated a single clean band of expected size [308 bp for PrP-Oct5 and 452 bp for PrP-Oct11a, consisting of 131 bp upstream of the octarepeats, the Oct5 (123 bp) or Oct11a (267 bp) octarepeats, and 54 bp downstream of the octarepeats], confirming the specificity of the PCR reactions (Figure 3A). Second, the PCR products, which contained molecules with either the wild type (input) octarepeats or mutant octarepeats due to PCR errors (termed PCR-mutant), were cloned and analyzed to determine the percentage of PCR-mutant molecules in the PCR products (Figure 2A). An aliquot of the PCR product was ligated into the pGEM-T vector and transformed into DH5α competent cells. After direct colony screening by PCR, the colonies that contained non-input octarepeats were cultured and plasmid DNAs were extracted and analyzed by *Spe*I and *Sac*II digestions to release the octarepeat insert(s). This analysis revealed that, for PCR products of PrP-Oct5, 6 out of 750 (0.8%) colonies contained mutant octarepeats (Figure 3B, Table 1). Sequencing confirmed that these colonies contained mutant octarepeats with various numbers of repeats (Figure 3B). One was an insertion mutant with 6 repeats (R1-R2-R2-R2-R3-R4). The other five were deletion mutants with 1–3 repeats, including two with 3-repeats of different sequences (R1-R2a-R4 and R1-R3-R4), one with 2-repeats (R1a-R4), and two with 1-repeat of different sequences (R4 and R14) (Figure 3B). Mutant repeat units (R14, R1a and R2a) were found in three mutant clones (for sequences see Figure 1), possibly due to homologous recombination between repeats as previously proposed [3].

**Table 1.Mutation:** Mutation rate of octarepeats during PCR or DNA replication in *E.coli.*

		Mutation Rate (%)^a^			
Input	Amplification	Prep 1^b^	Prep 2^b^	Prep 3^b^	Total
PrP-Oct5	PCR (Taq)	NA	NA	NA	6/750 (0.8%)-monomer
					1/750 (0.1%)-dimer
					7/750 (0.9%)-all
PrP-Oct11a	PCR (Taq)	NA	NA	NA	15/147 (10.2%)-monomer
					10/247 (6.8%)-dimer
					25/247 (17%)-all
PrP-Oct5	PCR (Pwo)	NA	NA	NA	6/882 (0.7%)-monomer
					1/882 (0.1%)-dimer
					7/882 (0.8%)-all
PrP-Oct11a	PCR (Pwo)	NA	NA	NA	18/301 (6.0%)-monomer
					3/301 (1.0%)-dimer
					21/301 (7.0%)-all
pOct5	DH5α	1/1717	1/1347	1/1160	3/4234 (0.07%)
pOct11b	DH5α	1/104	2/108	1/104	4/316 (1.3%)
pOct5	XL1-Red	1/160	2/152	1/160	4/472 (0.8%)
pOct11b	XL1-Red	1/32	1/40	2/60	4/132 (3.0%)

a Number of colonies with mutant octarepeats/total colonies screened; percentage in the parenthesis.

b Each of the three plasmid preparations was made from a single *E.coli* colony.

PCR products of PrP-Oct11a produced 15 out of 147 (10.2%) colonies with mutant octarepeats (Figure 3C, Table 1). All 15 mutant clones were deletion mutants containing 2–10 repeats, with one clone each of 9 and 10 repeats [R1-(R2)^5^-R3g-R3-R4 and R1-(R2)^6^-R3g-R3-R4], 2 clones with a 6-repeats of the same sequence (R1-R2-R2-R3g-R3-R4),3 clones with 5-repeats of two sequences (R1-R2-R3g-R3-R4 and R1-R2-R2-R3-R4), 2 clones with 4-repeats of different sequences (R1-R2-R3-R4 and R1-R3g-R2-R4), 2 clones with 3-repeats of different sequences (R1-R2-R4 and R1-R3-R4), and 4 clones with 2-repeats of 3 different sequences (R1-R4, R1a-R4, R1a-R14).

In addition, 1 out of 750 clones (0.13%) derived from PrP-Oct5 PCR products produced two fragments upon restriction digestion with *Sac*II and *Spe*I; one fragment was the same as the template Oct5 octarepeats (R1-R2-R2-R3-R4) whereas the other was a deletion mutant (R1a-R4) (Figure 3D). Further transformation with this clone did not lead to separation of the two fragments and digestion with a single-cut restriction enzyme produced two bands instead of the single band expected for plasmid monomers, indicating that this mutant clone is a plasmid dimer containing a wild type Oct5 insert and a deletion insert, which is referred to as a mutant dimer (Figure 2A). Similarly, 10 out of 147 clones (6.8%) derived from PrP-OCT11a PCR products were mutant dimers (Figure 3E), of which one was an insertion mutant [R1-(R2)^9^-R3g-R3-R4] and nine were deletion mutants, the latter including one 9-repeat mutant [R1-(R2)^5^-R3g-R3-R4], seven 6-repeat mutants with three different sequences [R1-(R2)^2^-R3g-R3-R4, R1-(R2)^3^-R3-R4, and R1-(R2)^2^-R3g-R3-R4c] (see the sequence of R4c in Figure 1A), and one 5-repeat mutant [R1-(R2)^3^-R3-R4] (Figure 3E). Further examination of non-mutant colonies showed that 2 out of 30 (6.7%) contained wild type plasmid dimers for PCR products of PrP-Oct5 while 19 out of 30 (63.3%) contained wild type plasmid dimers for PCR products of PrP-Oct11a, indicating that the majority of colonies with plasmid dimers contained only wild type (input) octarepeats as expected. In contrast, the empty pGEM-T vector by itself failed to produce detectable plasmid dimers, indicating that the presence of the octarepeat inserts is critical for promoting dimer formation and Oct11 is more effective in causing dimer formation than Oct5. The observation that mutant colonies contained only mutant plasmid dimers suggests that the mutant plasmid dimers were initially formed during the ligation reaction (Figure 2A) through an unknown mechanism. One possibility is that some “paired” molecules are formed via interactions between the octarepeat regions of a wild type (input) molecule and a wild type or PCR-mutant molecule during the PCR reactions, then ligation of two pGEM-T plasmid vector molecules to one “paired” molecules during the ligation reaction results in a plasmid dimer. Such inter-molecule interactions could be enhanced by the repeated denaturation, renaturation, and DNA replication cycles of the PCR reaction and by the high concentrations of the PCR product molecules in the later cycles of the PCR reaction. If, instead, the mutant plasmid dimers were formed during replication in *E.coli*, then most colonies with mutant plasmid dimers should contain a larger amount of the original plasmid. Following transformation each colony starts from a single cell containing a single plasmid molecule and, with the low mutation rate in *E.coli*, mutation during replication will most often occur in later rounds of plasmid replication, making the mutant plasmid a minority of the final plasmid DNA pool in the resulting colony. Therefore, the actual total PCR mutation rate should be the sum of monomer mutants and dimer mutants, which is 7 out of 750 (0.9%) for the PrP-Oct5 template and 25 out of 147 (17.0%) for the PrP-Oct11a template. These results indicate that the Taq PCR mutation rate for PrP-Oct11a is more than 18 times that of the wild type PrP-Oct5 template.

**Figure 4:**
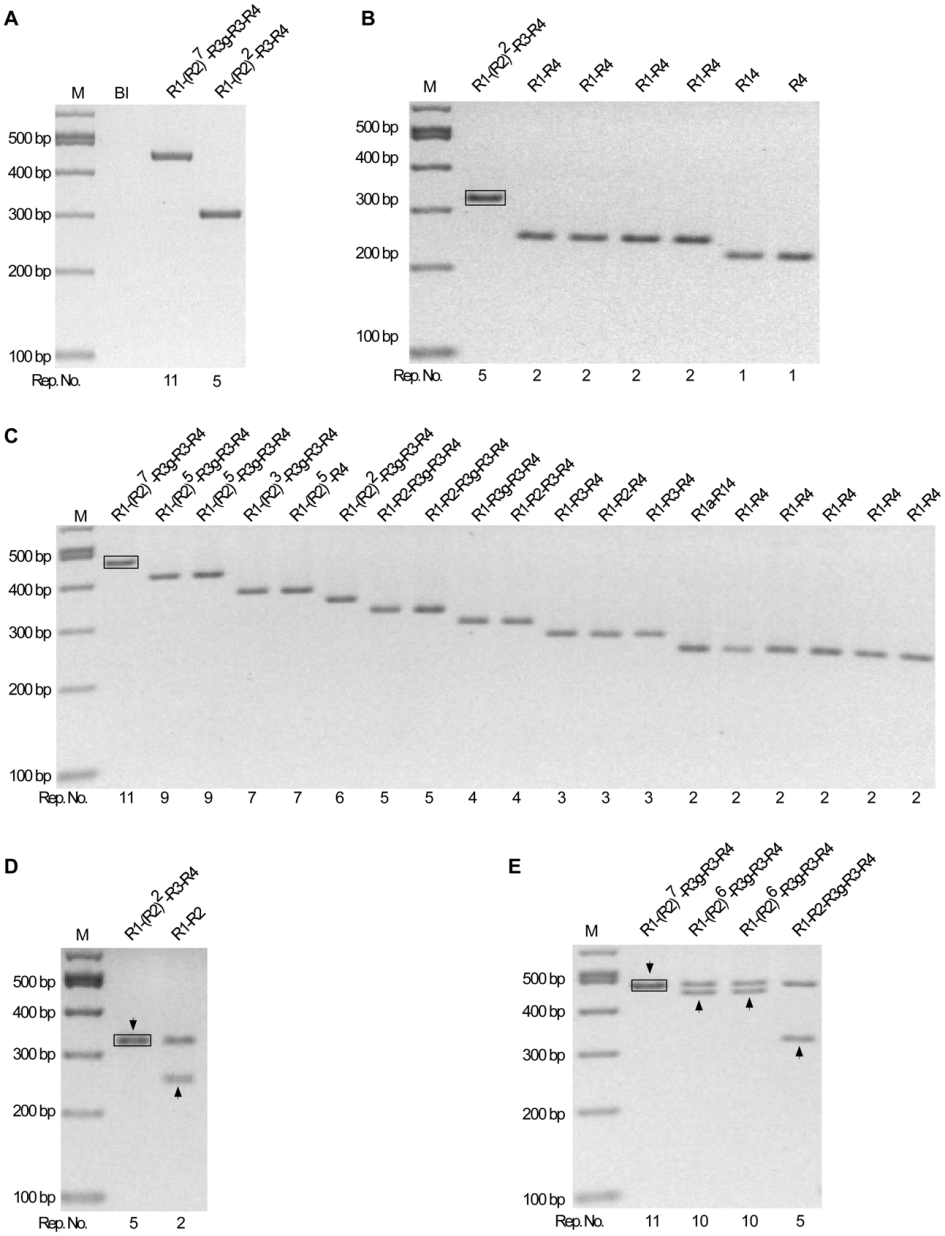
Instability of octarepeats during PCR amplification by Pwo polymerase. (A) PCR products from the PrP-Oct5 and PrP-Oct11a templates. The octarepeat regions PCR amplified by Pwo polymerase from PrP-Oct5 and PrP-Oct11a with primers HP20 and HP306r were cleaned up and separated on a 2% agarose gel. Bl, blank control. (B) Mutant octarepeat clones from PCR amplification of the PrP-Oct5 template: restriction analysis with *Sac*II and *Spe*I. Six mutant clones and one wild type clone are shown. The black box marks the template-sized Oct5 band from a non-mutant clone. (C) Mutant octarepeat clones from PCR amplification of the PrP-Oct11a template: restriction analysis with *Sac*II and *Spe*I. Same as in (B) except that PrPOct11a was the template DNA. Eighteen mutant clones and one wild type clone are shown. The black box marks the template-sized Oct11 band from a non-mutant clone. (D) A mutant octarepeat clone containing two octarepeat inserts from PCR amplification of PrP-Oct5. *Sac*II and *Spe*I digestion of this mutant plasmid clone produced two octarepeat inserts; one was the wild type Oct5 while the other was a 2-repeat deletion mutant (R1-R2). The arrowhead points to the band whose sequence is shown above the lane. The black box marks the template-sized Oct5 band from a non-mutant clone. (E) Mutant octarepeat clones containing two octarepeat inserts from PCR amplification of PrP-Oct11a. *Sac*II and *Spe*I digestion of the 3 mutant clones produced two octarepeat inserts; one was the 11-repeat parental Oct11a in all clones while the other was a mutant octarepeat sequence of varying sizes and sequences. The arrowhead points to the band whose sequence is shown above the lane. The black box marks the template-sized Oct11 band from a non-mutant clone. For all panels, the octarepeat sequence is indicated above each lane; Rep. No., number of repeats; M,100-bp DNA Ladder.

PCR products of PrP-Oct5 and PrP-Oct11a produced by the high fidelity Pwo polymerase were also similarly examined (Figure 4). PCR products of PrP-Oct5 produced 6 out of 882 (0.7%) colonies with mutant octarepeats of various repeat numbers (Table 1; Figure 4B). All six were deletion mutants with 1–2 repeats, including four with a 2-repeat (R1-R4), and two with 1-repeat of different sequences (R14 and R4) (Figure 4B). PCR products from the PrP-Oct11a template produced18 out of 301 (6.0%) colonies with mutant octarepeats (Figure 4C, Table 1). All 18 mutant clones were deletion mutants containing 2–9 octarepeats, including two clones with a 9-repeat of the same sequence [R1-(R2)^5^-R3g-R3-R4], 2 clones with a 7-repeat of different sequence [R1-(R2)^3^-R3g-R3-R4 and R1-(R2)^5^-R4], 1 clone with a 6-repeat (R1-R2-R2-R3g-R3-R4), 2 clones with a 5-repeat of the same sequence (R1-R2-R3g-R3-R4), 2 clones with a 4-repeat of different sequences (R1-R2-R3-R4 and R1-R3g-R3-R4), 3 clones with a 3-repeat of two different sequences (1 of R1-R2-R4, 2 of R1-R3-R4), and 6 clones with a 2-repeat of 2 different sequences (5 of R1-R4, 1 of R1a-R14) (Figure 4C). Rare clones containing mutant plasmid dimers were also found: 1 out of 882 clones from the PrP-Oct5 template contained Oct5 and R1-R2 (Figure 4D) while 3 out of 301 clones from the PrP-Oct11a template contained Oct11a and either R1-(R2)^6^-R3g-R3-R4 (2 clones) or R1-R2-R3g-R3-R4 (1 clone) (Figure 4E). Thus, the total Pwo PCR mutation rate is 0.8% (7 out of 882) for the PrP-Oct5 template and 7.0% (21 out of 301) for the PrP-Oct11a template.

In comparison to the results obtained using Taq polymerase, the Pwo octarepeat mutation rate is similar for the wild type human octarepeats Oct5 (0.8% vs 0.9%) but moderately lower for the insertion mutant Oct11a (7.0% vs 17.0%) (Table 1), indicating that the fidelity of the polymerase used has only limited influence on the PCR mutation rate of the octarepeat regions.

### Mutation of the octarepeat regions during DNA replication in DH5α *E.coli* cells

The instability of the octarepeat region was further evaluated during DNA replication *in vivo* in DH5α, a popular *E.coli* strain for molecular cloning procedures, which contains the *rec*A1 mutation that results in enhanced insert stability and the *end*A1 mutation that leads to improved plasmid quality and yield.

First, the octarepeat regions in PrP-Oct5 and PrP-Oct11b were amplified by PCR with primers HP20 and HP306r and subcloned into the pGEM-T vector and sequenced to obtain pOct5 and pOct11b (Figure 1B-C). The cloned pOct5 plasmid was used to transform competent DH5α cells, and 3 plasmid DNA samples were prepared, each from a single colony. Similarly, 3 plasmid DNA samples were prepared from pOct11b transformed DH5α. These plasmid DNA samples contained two types of plasmid molecules: those with just the original octarepeats and those with both the original octarepeats and mutant octarepeats resulting from DNA replication errors in *E.coli* (termed replication-mutant) (Figure 2B). Next, these plasmid DNA samples were used to transform competent DH5α cells. After direct colony PCR screening, colonies with replication-mutant octarepeats were picked for restriction analysis with *Sac*II and *Spe*I and sequencing. The percentage of replication-mutant plasmid molecules in the original plasmid samples was determined by counting the number of colonies containing replication-mutant octarepeats over the total number of colonies screened (Figure 2B), which serves as a measure of octarepeat mutation rate during DNA replication in DH5α.

**Figure 5:**
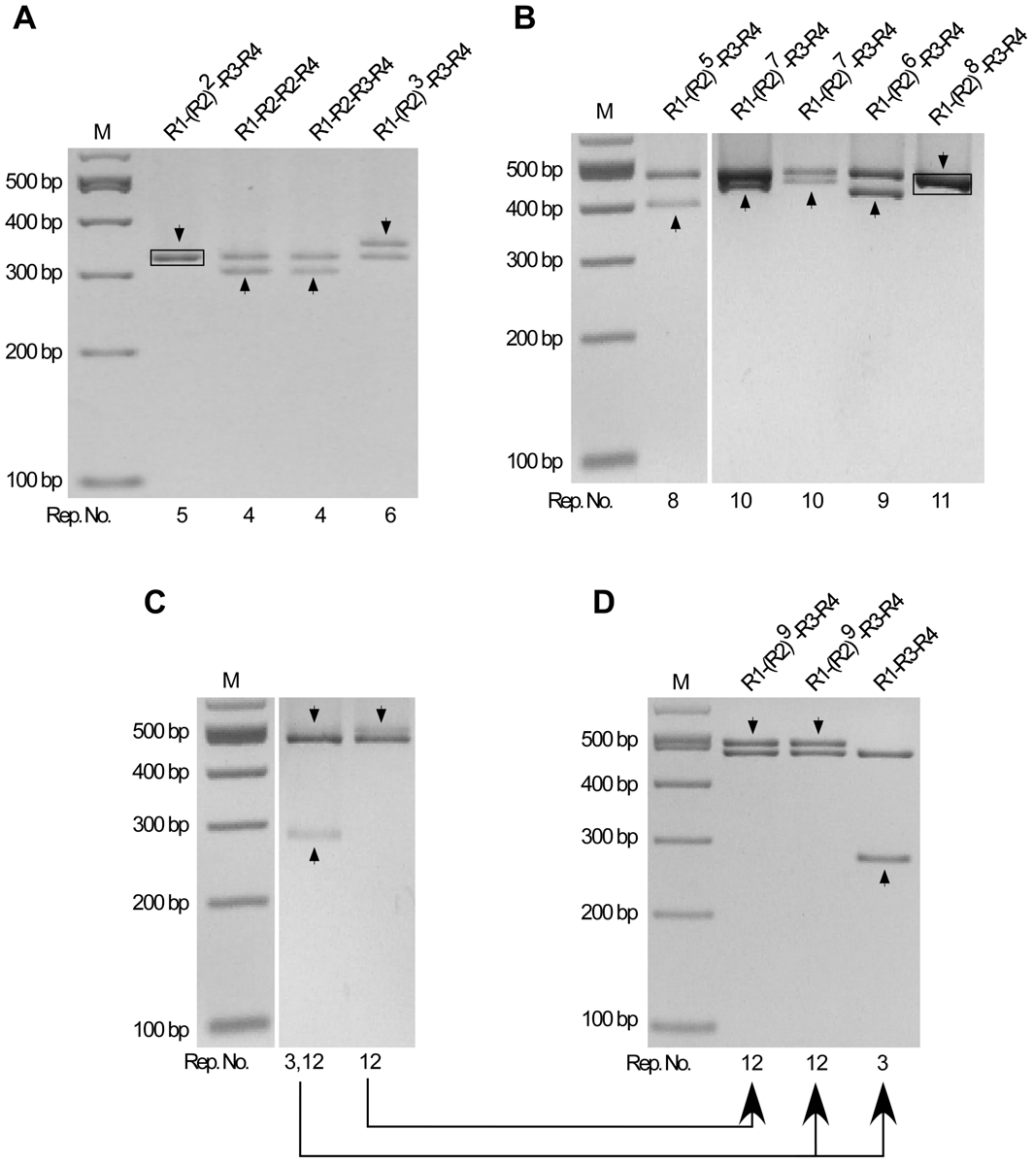
Instability of octarepeats during DNA replication in DH5α cells. (A) Mutant clones from replication of pOct5 in DH5α cells. pOct5 was transformed into DH5α. Plasmid DNAs were prepared from the resulting colonies, digested with *Sac*II and *Spe*I and separated on a 2% agarose gel. Shown are plasmid DNAs from 3 colonies that produced two octarepeat bands of equal molar ratio. (B) Mutant clones from replication of pOct11b in DH5α cells. pOct11b was transformed into DH5α. Plasmid DNAs were prepared from the resulting colonies, digested with *Sac*II and *Spe*I and separated on a 2% agarose gel. Shown are plasmid DNAs from 4 colonies that produced two octarepeat bands of equal molar ratio. (C–D) Unusual mutant clones from replication of pOct11b in DH5α cells. Shown are plasmid DNAs from two pOct11b-transformed DH5α colonies that produced 2–3 octarepeat bands upon digestion with *Sac*II and *Spe*I, of which the template-sized band is much stronger than the mutant bands (C). The unequal molar ratio of the octarepeat bands suggests the presence in these colonies of mixed plasmid DNA species where each species produced one of the octarepeat bands. Re-transformation of these plasmid DNAs into DH5α cells resulted in separation of the mixed plasmid DNA species and produced colonies that each contained only one plasmid DNA species as confirmed by restriction analysis and sequencing (D). For all panels, the octarepeat sequence is indicated above each lane, the arrowhead points to the band whose sequence is shown above the lane, and the black box marks the template-sized Oct5 or Oct11 band from a non-mutant clone. Rep. No., number of repeats; M,100-bp DNA Ladder.

For pOct5, of the 4234 colonies screened, only 3 (0.07%) contained replication-mutant octarepeats (Table 1). The three replication-mutant octarepeats were all different: one was an insertion mutant of 6-repeats [R1-(R2)^3^-R3-R4] while the other two were deletion mutants of different 4-repeat sequences (R1-R2-R2-R4 and R1-R2-R3-R4) (Figure 5A). Interestingly, all three colonies with replication-mutant octarepeats contained plasmids that produced two octarepeat insert bands after digestion with *Sac*II and *Spe*I (Figure 5A); sequencing revealed that one of the two insert bands contained the parental Oct5 octarepeats while the other harbored the replication-mutant octarepeats. Further transformation into DH5α with the three clones and restriction analysis confirmed that these clones were all plasmid dimers.

For pOct11b, 4 out of 316 DH5α colonies (1.3%) screened contained replication-mutant octarepeats, indicating a mutation rate that is 18 times higher than that of pOct5 in DH5α (Table 1). All were deletion mutants, with one 8-repeat [R1-(R2)^5^-R3-R4], one 9-repeat [R1-(R2)^6^-R3-R4] and two identical 10-repeat [R1-(R2)^7^-R3-R4] regions (Figure 5B). Again, all mutant plasmid DNAs were dimers that produced the parental Oct11 band in addition to the mutant octarepeat band (Figure 5B). There were also two colonies containing mixed plasmid DNAs that produced a strong Oct11 band plus 1–2 weaker mutant octarepeat bands (Figure 5C). Re-transformation of plasmid DNAs from these two colonies into DH5α led to two kinds of colonies, with the majority containing only pOct11b and a minority containing plasmids with just the newly generated mutant octarepeats. Sequencing confirmed that one of the two colonies contained two types of mutant plasmid DNAs, one had a 12-repeat [R1-(R2)^9^-R3-R4] and the other had a 3-repeat (R1-R3-R4); the other colony contained only one type of mutant plasmid with a 12-repeat [R1-(R2)^9^-R3-R4] (Figure 5D). We termed the mutant octarepeats in colonies with mixed plasmids “NEW replication-mutant” (Figure 2B) because these mutants were likely due to *de novo* mutations that were not present in the original plasmid DNA samples prepared after the first round of plasmid replication and cell division in *E.coli* (Figure 2B).

### Defective DNA mismatch repair augments octarepeat mutation in *E.coli* cells

DNA mismatch repair is involved in correcting DNA mutations, and aging leads to compromised DNA repair that may underlie many age-related diseases [14]–[22]. To assess the influence of DNA mismatch repair on octarepeat mutations, the mutation rates of pOct5 and pOct11b were examined in XL-1 Red *E.coli* cells, which have no functional mismatch repair genes since all three primary mismatch repair genes (mutS, mutD, mutT) are defective.

The same protocol used to analyze octarepeat mutation in DH5α cells was applied to pOct5 and pOct11b plasmid DNA samples prepared from XL-1 Red *E.coli* cells (Figure 2B).

**Figure 6:**
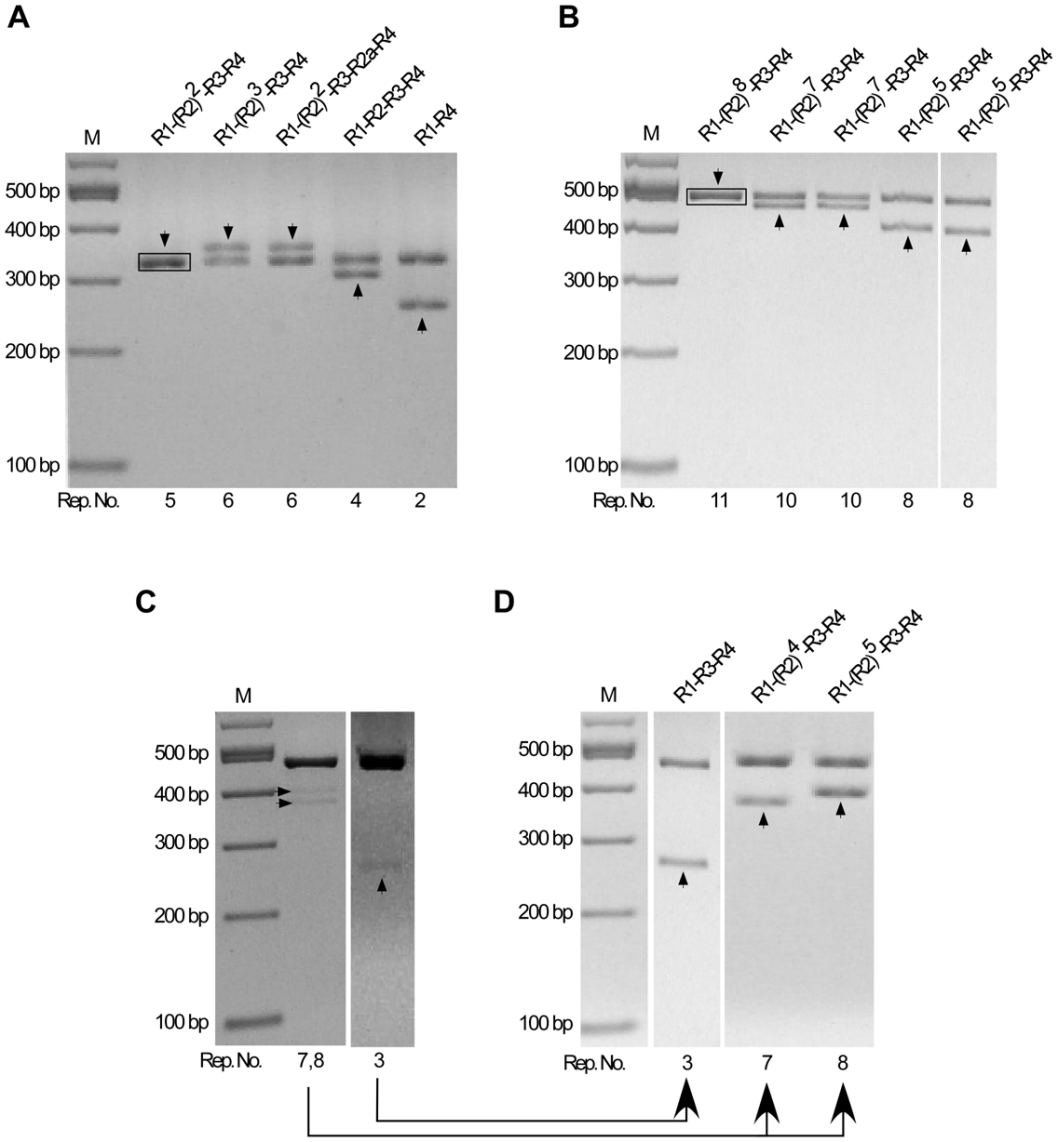
Instability of octarepeats during DNA replication in mismatch repair-deficient XL-1 Red cells. (A) Mutant clones from replication of pOct5 in XL-1 Red *E.coli* cells. Plasmid DNA samples were prepared from XL-1 Red colonies after transformation with pOct5 and re-transformed into DH5α cells. Plasmid DNAs from the resulting DH5α colonies were digested with *Sac*II and *Spe*I and separated on a 2% agarose gel. Shown are plasmid DNAs from 4 DH5α colonies that produced two octarepeat bands of equal molar ratio. (B) Mutant clones from replication of pOct11b in XL-1 Red *E.coli* cells. Same as in (A) except that pOct11b was used. Shown are plasmid DNAs from 4 colonies that produced two octarepeat bands of equal molar ratio. (C–D) Unusual mutant clones from replication of pOct11b in XL-1 Red *E.coli* cells. Shown are plasmid DNAs from two pOct11b-transformed XL-1 Red colonies that produced 2–3 octarepeat bands upon digestion with *Sac*II and *Spe*I, of which the template-sized band is much stronger than the mutant bands (C). The unequal molar ratio of the octarepeat bands suggests the presence of mixed plasmid DNA species in these colonies. Further transformation of these plasmid DNAs into DH5α cells resulted in separation of the mixed plasmid DNA species and produced colonies that each contained only one of the plasmid DNA species as confirmed by restriction analysis and sequencing (D). For all panels, the octarepeat sequence is indicated above each lane, the arrowhead points to the band whose sequence is shown above the lane, and the black box marks the template-sized Oct5 or Oct11 band from a non-mutant clone. Rep. No., number of repeats; M,100-bp DNA Ladder.

Plasmid DNAs were prepared from single colonies after transformation of competent XL-1 Red cells with pOct5 or pOct11b. After restriction analysis with *Sac*II and *Spe*I, single-colony plasmid DNA minipreps from XL-1 Red, three each for pOct5 and pOct11b that showed no visible non-parental octarepeat band, were selected as the input plasmid DNA preparations for further transformation in DH5α cells to measure the percentage of octarepeat mutants after propagation of pOct5 and pOct11b in XL-1 Red cells. This pre-selection of plasmid DNA preparations from XL-1 Red cells is important because plasmid DNA preparations that produced a visible mutant (non-input) octarepeat band(s) would lead to an overestimate of the mutation rate. For pOct5, of the 472 DH5α colonies screened, only 4 (0.8%) contained mutant octarepeats (Table 1), including two distinct insertion mutants with a 6-repeat [R1-(R2)^3^- R3-R4 and R1-R2-R2-R3-R2a-R4] and two deletion mutants with a 4-repeat (R1-R2-R3-R4) and a 2-repeat (R1-R4), respectively (Figure 6A). For pOct11b, of the 132 DH5α colonies screened, 4 (3.0%) contained mutant octarepeats (Table 1). The four mutant octarepeats were all deletion mutants, including two identical 10-repeats [R1-(R2)^7^-R3-R4] and two identical 8-repeats [R1-(R2)^5^-R3-R4] (Figure 6B). In addition, similar to the findings for DH5α-replicated pOct11b, 2 of the 132 colonies from XL-1 Red-replicated pOct11b contained NEW replication-mutant plasmid dimers that produced the parental Oct11 band plus 1-2 weaker mutant octarepeat bands upon digestion with *Spe*I and *Sac*II (Figure 6C). Subsequent separation by further transformation in DH5α cells and sequencing confirmed that each of the weaker mutant octarepeat band represented a plasmid dimer species containing the parental Oct11b insert plus a mutant octarepeat insert, the latter includes a 3-repeat (R1-R3-R4), a 7-repeat [R1-(R2)^4^-R3-R4] and an 8-repeat [R1-(R2)^5^-R3-R4] (Figure 6D).

The octarepeat mutation rates are significantly higher in XL-1 Red cells than in DH5α cells for pOct5 (0.8% vs. 0.07%, p = 0.003 by Fisher's exact test), but the difference for pOct11b is not statistically significant (3.0% vs. 1.3%, p = 0.183 by Fisher's exact test). These results indicate that defective mismatch repair could dramatically increase the mutation rate for the wild type Oct5 octarepeats.

### Replication-mutant plasmids are all head-to-head dimers

**Figure 7:**
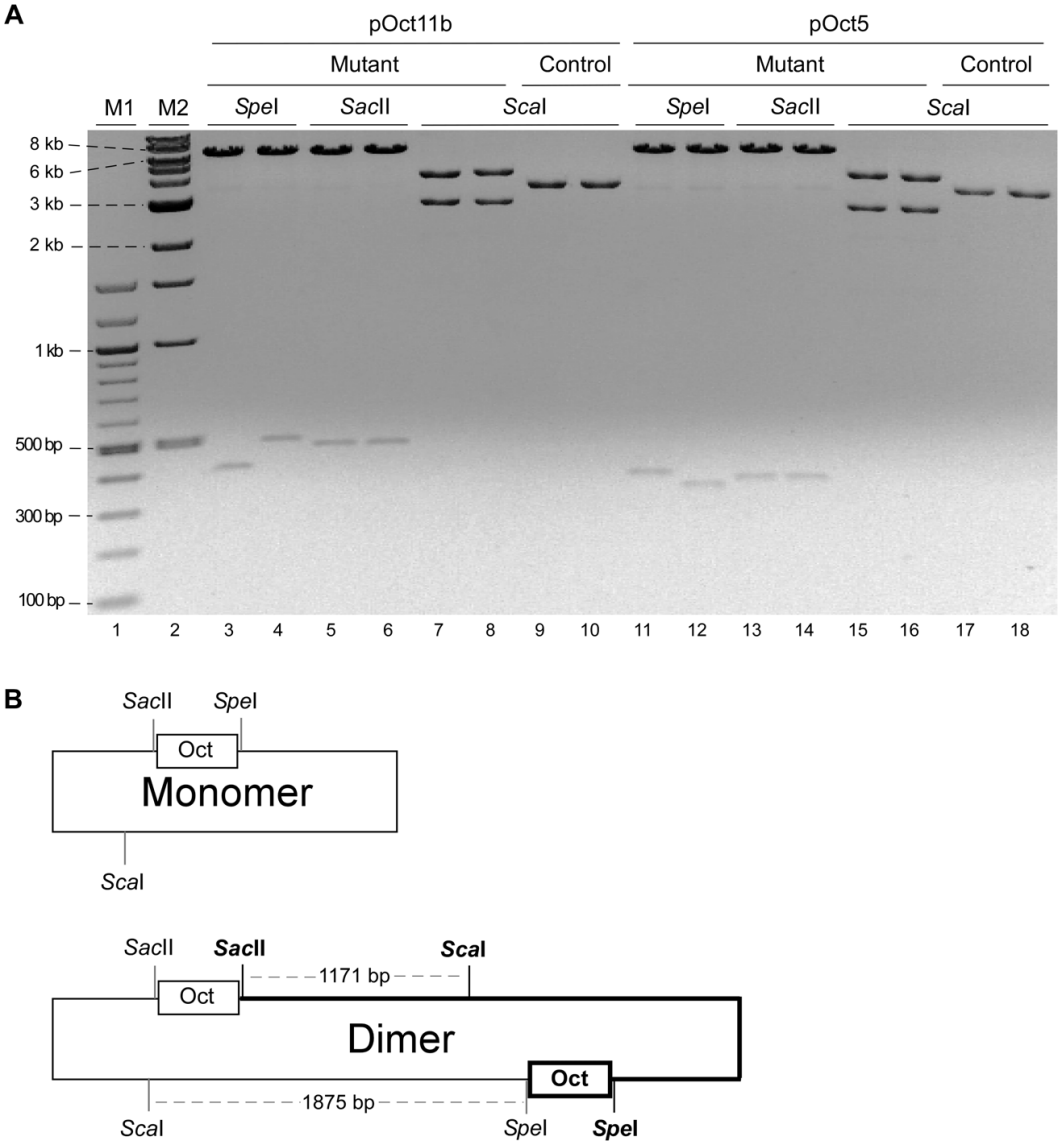
Mutant plasmids from octarepeat replication in DH5α are all head-to-head dimers. (A) Restriction analysis of replication-mutant plasmids with *Spe*I, *Sac*II and *Sca*I. pOct5 or pOct11b were transformed into DH5α. Plasmid DNAs were prepared from two mutant colonies and two control (non-mutant) colonies each for pOct5 and pOct11b, digested with *Spe*I, *Sac*II or *Sca*I, and separated by agarose gel electrophoresis. All mutant colonies appear to contain a minute amount of monomer plasmids. M1, 100-bp DNA ladder; M2, 1-kb DNA ladder. (B) Diagram of the head-to-head plasmid dimers. The top panel depicts the parental plasmid monomer; the bottom panel depicts the dimer where the newly generated monomer unit is highlighted in thicker lines. The boxes denote the octarepeat inserts.

After propagation of pOct5 and pOct11b in DH5α or XL-1 Red cells, all resulting mutant plasmids were plasmid dimers containing the parental octarepeats plus a mutant octarepeat sequence (Figures 5–6). To dissect the structures of these plasmid dimers, plasmid DNAs were prepared from two mutant colonies (containing only mutant plasmid dimer) and two control colonies (containing only wild type plasmid monomer) after transformation of pOct5 into DH5α, digested with *Sac*II, *Spe*I, or *Sca*I, and subjected to agarose gel electrophoresis (Figure 7A). Two mutant plasmid samples derived from replication of pOct11b in DH5α were similarly examined (Figure 7A). Upon *Sca*I digestion, the control plasmids produced a single band with the expected size corresponding to a monomer (lanes 9, 10 and 17, 18 in Figure 7A), whereas the mutant plasmids produced two bands whose combined size equals to a dimer (lanes 7, 8 and 15, 16 in Figure 7A). PCR analysis of the DNA recovered from these two bands revealed that the smaller band contained the parental octarepeats while the bigger band contained the mutant octarepeats. Upon *Sac*II digestion, the mutant plasmids produced two bands (lanes 5, 6 and 13, 14 in Figure 7A); the smaller band (∼0.4–0.5 kb) corresponds to the parental octarepeat insert while the larger band (∼6 kb) corresponds to the plasmid dimer backbone containing the mutant octarepeat insert. Upon *Spe*I digestion, the mutant pOct11b and pOct5 plasmids also produced two bands (lanes 3, 4 and 11, 12 in Figure 7A); the smaller band (∼0.3–0.4 kb) corresponds to the mutant octarepeat insert while the larger band (∼6 kb) corresponds to the plasmid dimer backbone containing the parental octarepeat insert.

These results revealed that all the mutant plasmids were head-to-head dimers where one monomer unit contained the parental octarepeats while the other monomer unit contained the replication-mutant octarepeats (Figure 7B). Examination of mutant plasmid samples derived from replication of pOct5 and pOct11b in XL-1 Red cells gave the same result (data not shown).

## Discussion

Little is known about the etiology of sCJD in humans. In the present study, we showed that the wild type 5-repeat octarepeat region (Oct5) in the human *PRNP* gene is unstable during PCR amplifications with a mutate rate of 0.9%. The insertion mutant Oct11a was more unstable with a mutation rate of 17.0% during PCR, 18 times greater than that of Oct5 (Table 1). Mutation analysis of Oct5 and Oct11b during DNA replication in wild type *E.coli* (DH5α) revealed octarepeat mutation rates of 0.07% and 1.3%, respectively. The spontaneous DNA mutation rate based on the frequency of *lacI* mutants was 0.00013% in the wild type NR9102 *E.coli* strain [23], which is more than 500 times lower than the spontaneous octarepeat mutation rates we observed in the DH5α strain. Furthermore, in the XL-1 Red *E.coli* cells where all three major DNA mismatch repair genes were defective, Oct5 had a mutation rate of 0.8%, 11 times higher than that in DH5α; Oct11b had a mutation rate of 3.0%, which is also higher than the 1.3% mutation rate found in DH5α although the difference did not reach statistical significance. These results demonstrate that the human PrP octarepeat region is inherently unstable during DNA replication, octarepeat insertion mutants are even less stable, and the instability of octarepeats is exacerbated by compromised mismatch repair. Given the established link of octarepeat mutations to familial prion diseases [1], [24], our data support an octarepeat somatic mutation-based etiology for sCJD as follows: the instability of the octarepeat region leads to accumulation of somatic octarepeat mutations in brain cells during development and aging, and the instability is augmented by compromised DNA mismatch repair in aged cells; eventually some of the octarepeat mutation-containing brain cells start spontaneous *de novo* prion formation and replication to initiate sCJD. The heterogeneous phenotypes associated with various octarepeat mutations [1] could explain the phenotypic diversity of sCJD. A large number of point mutations in the *PRNP* coding region have also been linked to familial prion diseases in humans [1]. Consequently, somatic point mutation in non-repeat *PRNP* coding regions in human brain cells could also be a potential cause for sCJD and needs to be examined in the future. Other remaining questions include: 1) Are octarepeat sequences from PrPs of other species also unstable? 2) Do human octarepeat sequences promote higher mutation rates in flanking regions? 3) Are other repeat sequences in non-PrP genes unstable as well? These are all important areas for future investigations.

Taq polymerase is of low fidelity and only 17 PCR cycles were used in experiments to measure the octarepeat PCR mutation rate. However, the commonly cited fidelity rates for PCR polymerases are based on measurement of point-mutation errors, which may have very different mechanism from that of repeat expansion or contraction. To evaluate the impact of the polymerase fidelity on the PCR mutation rate of the octarepeat regions, we repeated the PCR experiments with the high fidelity Pwo polymerase (Figure 4), whose point mutation rate is 18-fold lower than that of Taq polymerase (http://www.roche-applied-science.com). Our results indicate that polymerase fidelity, as assessed on point mutations, has very limited influence on the mutation rate of the wild type human octarepeats (Table 1), suggesting that the mechanism for repeat expansion and contraction are indeed different from those of point mutations.

The mutation rate of the wild type octarepeat sequence is still below 1% in both DH5α and XL-1 Red cells, which is consistent with the rarity of sCJD. However, in theory, a single brain cell containing a somatic octarepeat mutation could generate the first prion seed, thereby initiating sCJD. Since there are billions of neurons and glial cells in a human brain, even a mutation rate of 0.001% will translate into tens of thousands of brain cells carrying an octarepeat mutation. Nevertheless, the occurrence of sCJD is very rare because spontaneous *de novo* prion conversion must still be a rare event in cells harboring somatic octarepeat mutation. Even in familial octarepeat mutation cases in which all cells carry the mutation there is a strong age-dependent component [1]. The majority of mutations we observed in PCR and DNA replication in *E.coli* were deletions rather than insertions, but octarepeat deletions are less commonly associated with familial prion disease [1]. On one hand, the observed dominance of deletions during PCR and DNA replication in *E.coli* is consistent with the rarity of sCJD. On the other hand, it is important to note that the proportion of octarepeat insertion mutants in human brain is likely much higher than what we observed in *E.coli*, because it has been reported that repeat contraction due to deletion biases is common in bacteria while eukaryotes generally experience unbiased mutation or a bias towards insertion or repeat expansion [25]. In addition, the highly increased mutation rate for the octarepeat insertion mutants does not explain sCJD, but it may underscore the fact that the wild type octarepeat region in humans has only 5 repeat units.

Reports of somatic point mutations in presenilin 1 gene in a case of sporadic early onset Alzheimer's disease [11] and in the *PRNP* gene in a sCJD case [26] suggest that somatic point mutations in other coding positions in *PRNP* may also lead to sCJD. The next challenge is to set up sensitive and reliable assays for direct measurement of somatic mutation rates of octarepeats in neurons and other brain cells from brain tissues of normal individuals and sCJD subjects. It is worth noting that the observed high rate of octarepeat mutation during PCR amplification indicates that PCR should not be used to assess octarepeat mutation levels in human genomic DNAs if the mutation level is low. Indeed, probably all PCR-based data showing low levels of mutation in any extensive repeat sequence should be interpreted with caution. Fortunately, since all octarepeat mutants resulting from plasmid DNA replication in *E.coli* are distinct and reside only in head-to-head plasmid dimers (Figure 7), *E.coli* can still be used to directly quantify low levels of octarepeat mutants in a genomic DNA sample if there is no prior PCR amplification.

**Figure 8:**
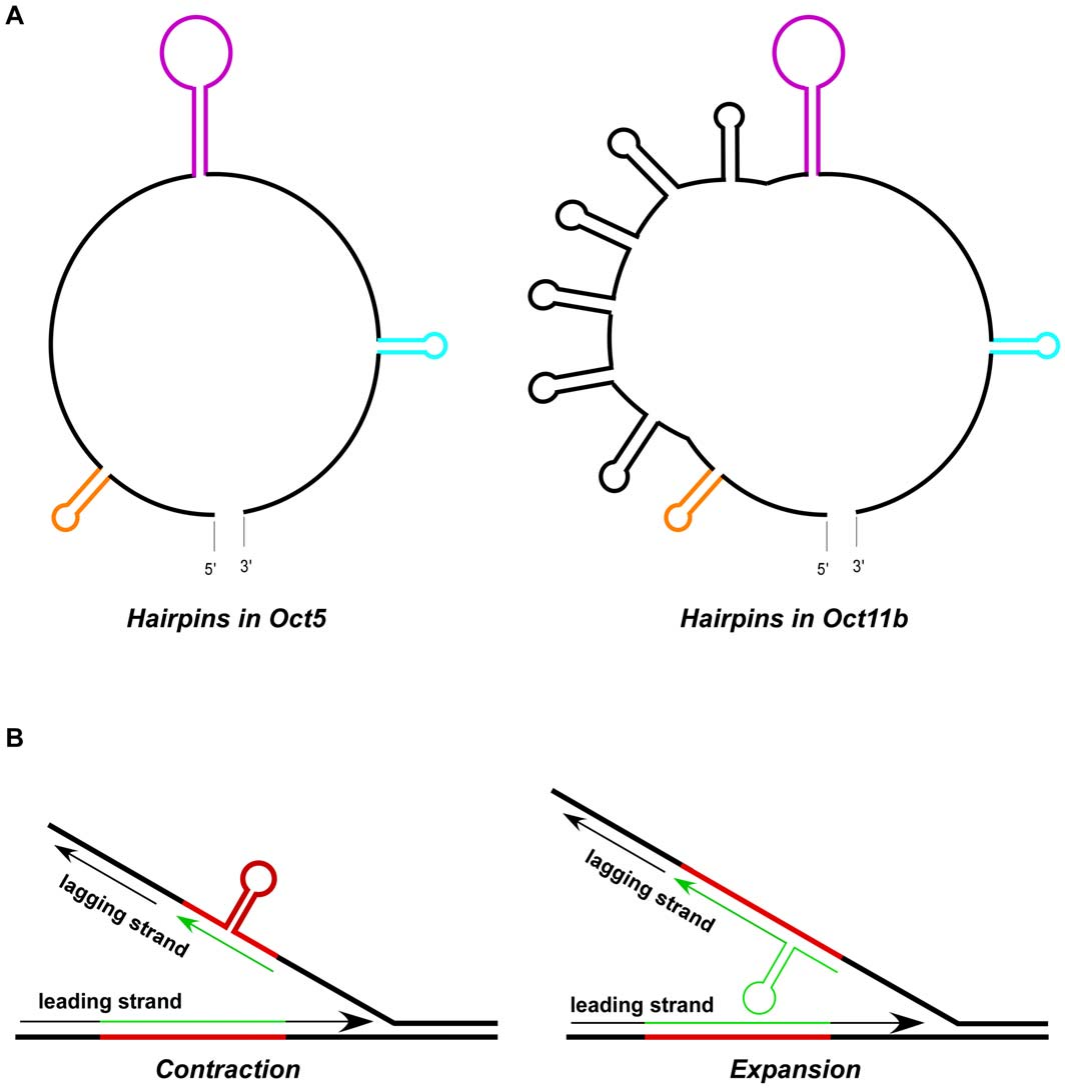
A hairpin structure-based model for mutation of octarepeats. (A) Diagram of hairpin structures of Oct5 and Oct11b as predicted by mfold. The likely secondary structures of Oct5 and Oct11b octarepeat sequences were examined with the online mfold program (http://mfold.rna.albany.edu/?q=mfold/DNA-Folding-Form). Only the most stable structure for each sequence as predicted by mfold at the PCR annealing condition (50 mM Na^+^, 1.5 mM Mg^2+^, 58.5°C) is depicted, but the *E.coli* condition (170 mM Na^+^, 37°C) yielded similar results. The three colored hairpins represent the common ones shared by Oct5 and Oct11b sequences; the insertion of 6 extra repeats in Oct11b led to 6 additional hairpins (in black) that are identical to the first hairpin (in orange). (B) Hairpin-based slippage model for contraction or expansion of the octarepeats. The parental DNA strands are in thick lines and the newly synthesized DNA strands (leading strand or lagging stand) are in thin lines. The red lines denote the octarepeat region on parental DNA strands while green lines denote the octarepeat region on the newly synthesized DNA strands; hairpin formation on the parental DNA strand leads to contraction whereas hairpin formation on the lagging strand being synthesized results in expansion. Adopted from Figure 3 in [29].

Significantly, all mutant octarepeats resulting from plasmid DNA replication in *E.coli* were contained in head-to-head plasmid dimers. Cruciform-dumbbell structures (hairpins on both DNA strands) have been proposed to facilitate head-to-head plasmid dimer formation in *E.coli*, resulting from cleavage of the cruciform structure followed by replication [27], [28]. Analysis with the mfold program (http://mfold.rna.albany.edu/?q=mfold/DNA-Folding-Form) reveals that both DNA strands of Oct5 and Oct 11 octarepeats can form multiple stable hairpin structures even under PCR reaction conditions (Figure 8A). These observations suggest that the octarepeat region tends to form hairpin structures on one or both DNA strands during DNA replication, DNA repair or transcription. The hairpin structures in turn promote mutation of the octarepeats through a mechanism like DNA polymerase slippage [29] (Figure 8B), and mismatch repair proteins play a significant role in the mutation process. In addition, aging is known to contribute to somatic mutations [30]–[33]. One major mechanism for age-related somatic mutations is erroneous DNA repair after DNA damages in aged cells [19], [31]. In aged human brains, compromise of the mismatch repair system may further enhance octarepeat mutation and increase the chances of spontaneous *de novo* formation of sCJD prions; the *de novo* prion agents may then spread to surrounding cells initiating sCJD.

The PrP octarepeat region has significant biological functions. The octapeptide repeats bind copper [34]–[38] and other divalent cations such as zinc [39] in a pH-sensitive manner, and copper and iron metabolism is abnormal in PrP-null or prion infected mice [40]–[43]. The octapeptide repeat region is also crucial for metal ion-induced endocytosis of the prion protein [44], [45], prevention of Purkinje cell death in a transgenic mouse model [46], and PrP-mediated inhibition of Bax-induced cell death in human primary neurons [47]. Some reports have shed light on how octarepeat mutations may cause genetic prion diseases in humans. PrP with expanded octarepeats due to insertion mutations were more prone to aggregate and the extent of aggregation was proportional to the number of repeats [48]–[50]. The size of the octarepeat region also affects PrP conversion by PrP^Sc^ [51]–[53]. The increased propensity towards aggregation of PrP with expanded octarepeats may underlie inherited prion diseases caused by mutations of the octarepeat region. Moreover, brain homogenates from human subjects with 5, 7 or 8 extra octapeptide repeats transmitted the disease to non-human primates [3], [54]. However, the transmissibility of the nine octapeptide repeat insertion mutant has not been tested and a transgenic mouse model expressing mouse PrP with a 14-repeat octarepeat region developed spontaneous, but non-transmissible, neurodegenerative disease [55], [56].

Tandem repeats, including microsatellites (simple tandem repeats of 2–8 nucleotides) and minisatellites (variable number of tandem repeats of usually 7–100 nucleotides that span 500–20,000 bp) are common in eukaryotic genes and promoters [57] and they mutate at high frequencies in both germ-line cells and somatic cells [30], [32], [58]–[61]. Intergenerational and somatic instability of repeat sequences has been convincingly associated with neurodegenerative diseases. Over 20 unstable microsatellite repeats consisting of repeats of 3–5 nucleotides have been linked to neurological diseases, including the CGG repeats in the FMR1 gene for fragile X syndrome, the CAG repeats in the androgen receptor for Kennedy's disease, the CAG repeats in the huntingtin gene for Huntington's disease (HD), the CTG repeats in the DMPK gene for myotonic dystrophy type 1, the CCTG repeats in the ZNF9 gene for myotonic dystrophy type 2, and the ATTCT repeats in the ATXN10 gene for spinocerebellar ataxia type 10 [62] . Many of the microsatellite repeats exhibit significant tissue-specific somatic instability. The CTG repeat in the DMPK gene of myotonic dystrophy type 1 patients is highly unstable in muscle cells [63], [64]. The CAG repeat region in the huntingtin gene also exhibits high levels of mosaicism in HD neurons/brains [8]–[10]. Moreover, the CAG repeat expansion in HD brains was reported to occur prior to clinical disease onset and become more pronounced over time [12]. The longer the repeat length of the somatically expanded CAG repeats, the earlier the age of disease onset [65]. In general, longer repeats are associated with more severe instability, which was confirmed in transgenic mouse models expressing CTG repeats of various lengths [66]. Germ-line and somatic minisatellite instability is also common in the human genome [58], [59], [67], [68]. The instability of the *PRNP* octarepeat region, which does not qualify as a minisatellite due to its limited size and contains mostly imperfect tandem repeats, suggests that somatic instability of less dramatic tandem repeats in functional genes may be more common than thought and their potential relationship to development of sporadic diseases should be examined more closely.

## Materials and Methods

### Ethics statement

The human brain tissue samples were obtained from the National Prion Disease Pathology Surveillance Center at Case Western Reserve University, Ohio. All samples are from deceased subjects, and personal information is limited to age, sex, symptoms, neuropathology, and classification of prion disease. These samples have been classified as “not human subjects” by the University Hospitals Case Medical Center Institutional Review Board since our research meets criteria for exemption under Federal regulations 45 CFR 46.102 (f).

### Genomic DNA extraction

Frozen human brain tissues were provided by the National Prion Disease Pathology Surveillance Center at Case Western Reserve University. Brain tissue (10–20 mg) was incubated at 55°C overnight in 560 µl of lysis buffer (50 mM Tris-Cl, 0.1 M EDTA,1% SDS,100 mM NaCl, 400 µg/ml of proteinase K (Roche, IN, USA), and genomic DNA was extracted using a standard phenol/chloroform protocol, dissolved in water and stored at−20°C in multiple aliquots for further experiments.

### Preparation of PCR templates (PrP-Oct5 and PrP-Oct11a) and plasmids (pOct5, pOct11b) for mutation analysis

Genomic DNA from one subject with wild type PrP-129MM was used to clone by PCR the wild type human PrP ORF template, which is named PrP-Oct5 for the five-repeat octarepeats (Figure 1B). Similarly, genomic DNAs from two subjects with PrP-129MM and different 11-repeat mutant alleles were used to clone by PCR the insertion mutant human PrP ORF templates, named PrP-Oct11a and PrP-Oct11b, respectively (Figure 1C). The human PrP ORF was amplified by PCR with a Mastercycler thermal cycler (Eppendorf, NY, USA) in a 50 µl reaction containing 50 ng human genomic DNA, 200 µM dNTPs (each), 1.5 mM MgCl_2_, 0.4 µM each of primers 42F (CATAACTTAGGGTCACATTTGTCC) and 45R (CCAGATTAACCAA-TGGTTATTTGC), and 5 units of Taq DNA polymerase (Roche, IN, USA). The PCR cycles were: 94°C for 2 min; 94°C for 30 sec, 58.5°C for 30 sec and 72°C for 90 sec for 31 cycles; 72°C for 7 min. All primers used in this study were HPLC-purified (Invitrogen, CA, USA). The A260/280 ratios of DNA samples in this study were 1.8–2.0 as assayed with a Nanovue spectrophotometer (GE Healthcare, NJ, USA). The expected PCR products were 1265 bp for PrP-Oct5 and 1409 bp for PrP-Oct11a and PrP-Oct11b, which include 232 bp upstream of the ATG initiation codon, the PrP ORF (762 bp for PrP-Oct5 and 906 bp for PrP-Oct11a/b), and 271 bp downstream of the stop codon. The PCR amplified PrP ORFs were cloned into the pGEM-T vector (Promega, WI, USA) (see below) and the sequences confirmed by automated sequencing using primers HP306f (GAGTAAGCCAAAAAC-CAACATG) and HP432r (CGATAGTAACGGTCCTCATAG). The PrP-Oct5 clone had the expected 5 wild type repeats (R1-R2-R2-R3-R4); the PrP-Oct11a clone had R1-(R2)^7^-R3g-R3-R4 and the PrP-Oct11b clone had R1-(R2)^8^-R3-R4 where (R2)^7^ and (R2)^8^ denote 7 and 8 consecutive R2 repeats, respectively (Figure 1A). Cloned PrP-Oct5 and PrP-Oct11a were used as the templates for PCR mutation analysis. The octarepeat region of PrP-Oct5, PrP-Oct11a and PrP-Oct11b were subcloned into pGEM-T after PCR amplification with primers HP20 (GGATGCTGGTTCTCT-TTGTG) and HP306r (CATGTTGGTTTTTGGCTTACTC) and confirmed again by sequencing to obtain pOct5, pOct11a and pOct11b, respectively (Figure 1B-C). pOct5 and pOct11b were used for octarepeat mutation analysis in *E.coli*.

### Mutation detection in PCR products or plasmid DNAs replicated in *E.coli*

The octarepeat mutation rates in PCR products or plasmid DNAs replicated in *E.coli* were measured as depicted in Figure 2. To measure the mutation rate in the octarepeat region during PCR amplification, the octarepeat region was amplified by PCR from PrP-Oct5 or PrP-Oct11a with primers HP20 and HP306r and either Taq polymerase (Roche, IN, USA) or Pwo polymerase (Roche, IN, USA). The PCR products were treated with the Wizard PCR clean-up kit (Promega, WI, USA), ligated to pGEM-T (for Pwo-amplified products, A-tailing was done first with Taq DNA polymerase in PCR buffer with 0.2 mM dATP) and transformed into competent DH5α cells (New England Biolabs, MA, USA) on LB-agar plates with X-gal. Direct colony PCR screening was conducted as follows. Individual white and light blue colonies were directly picked by pipette tips into 20 µl of PCR reaction mix [200 µM dNTPs (each), 1×PCR buffer containing 1.5 mM MgCl_2_, 0.4 µM each of primers, and 2 units of Taq DNA polymerase] and subjected to PCR with primers HP50F (GTGACCTGGGCCTCTGCAAG) and HP293R (CTTACTCGGCTTGTTCCACT) as follows: 94°C for 2 min; 94°C for 30 sec, 66.5°C for 30 sec and 72°C for 60 sec for 17 cycles; 72°C for 7 min. The PCR products were separated on 2% agarose gels containing ethidium bromide. Sometimes bacteria picked from 2–5 colonies were pooled for one PCR reaction to increase the screening throughput; if non-template sized band(s) was found for a pooled sample, each of the 2–5 colonies was then picked separately and plasmid DNA extracted and examined to determine the mutant-containing colony. The mutant-containing colonies were grown overnight in LB medium, and plasmid DNAs were extracted using a plasmid DNA miniprep kit (Promega, WI, USA) and subjected to double digestion with restriction enzymes *Sac*II and *Spe*I (New England Biolabs, MA, USA); the released octarepeat insert band(s) was recovered and subjected to automated sequencing. The 100-bp DNA ladder and 1-Kb DNA ladder (New England Biolabs, MA, USA) were used as markers for agarose gel electrophoresis. Colonies that contained mutant (non-input) octarepeats were counted as octarepeat mutants. The mutation rate was calculated as the number of octarepeat mutant colonies over the total number of colonies screened.

To measure the mutation rate of octarepeats during DNA replication in wild type *E.coli* (DH5α), pOct5 or pOct11b was transformed into DH5α. Plasmid DNA prepared from a single DH5α colony from transformation with pOct5 or pOct11b was used to as the input plasmid preparation. The input plasmid preparation was transformed into DH5α, and the resulting colonies were subjected to PCR screening. The colonies that produced a mutant (non-input) octarepeat band(s) during the PCR screen were subjected to overnight culture, plasmid DNA extraction, restriction enzyme analysis and sequencing. The mutation rate was calculated as the number of DH5α colonies containing non-input octarepeats over the total number of DH5α colonies screened. Three input pOct5 DNA preparations and three input pOct11b preparations, each extracted from a single DH5α colony, were examined in this fashion, and the average mutation rate calculated (Table 1).

Similar protocol was applied to estimating the mutation rates of Oct5 and Oct11b in the DNA repair-deficient XL-1 Red *E.coli* cells (Stratagene, CA, USA). pOct5 or pOct11b plasmid DNA was transformed into competent XL-1 Red cells. Plasmid DNA minipreps were obtained from single XL-1 Red colony and examined by restriction analysis with *Sac*II and *Spe*I. Three plasmid minipreps each for pOct5 and pOct11b that did not show visible mutant (non-parental) octarepeat insert band on agarose gels were selected as the input plasmid samples, which were used for further transformation into DH5α and octarepeat mutant screening as described above.

### Sequencing and alignment

Plasmid DNAs and gel-purified PCR products or restriction digestion fragments were subject to automated sequencing and the resulting sequences were aligned with wild type human PrP sequence using LALIGN (http://xylian.igh.cnrs.fr/bin/lalign-guess.cgi).

### Mfold analysis

Octarepeat sequences (Oct5 and Oct11) were subjected to the online DNA mfold analysis (http://mfold.rna.albany.edu/?q=mfold/DNA-Folding-Form) to predict potential secondary structures. The folding parameters for PCR conditions were 50 mM Na^+^ and 1.5 mM Mg^2+^ at the three PCR temperatures: 94°C (denaturation), 58.5°C (annealing), or 72°C (extension); the folding parameters for *E.coli* were 170 mM Na^+^ at 37°C.

### Statistics analysis

Statistical significance was evaluated by Fisher's exact test (1-sided) with the SPSS 13.0 software (SPSS Int.).
